# Comparative chloroplast genomics of Cypripedioideae: structural divergence, adaptive evolution, and phylogenomic insights

**DOI:** 10.3389/fpls.2025.1723281

**Published:** 2026-02-04

**Authors:** Xiaoyan Zhao, Xiaoxiao Lan, Zishuo Wang, Dong Li, Wei Sun, Yunpeng Gai, Bing Liu

**Affiliations:** 1 School of Grassland Science, Beijing Forestry University, Beijing, China; 2 School of Landscape Architecture and Horticulture, Yangzhou Polytechnic University, Yangzhou, China

**Keywords:** adaptive evolution, chloroplast genome, codon usage bias, Cypripedioideae, ecological adaptation, natural selection, phylogenomics, structural variation

## Abstract

The Cypripedioideae subfamily (slipper orchids) exhibits remarkable ecological diversity, spanning temperate to tropical habitats, yet the molecular mechanisms underlying its adaptive radiation remain poorly understood. Chloroplast genomes, with their critical roles in photosynthesis and conserved structure, offer a powerful system to investigate how evolutionary forces shape genomic adaptation. This study presents the most comprehensive analysis to date of codon usage bias (CUB) and structural evolution in Cypripedioideae chloroplast genomes, evaluating the relative contributions of mutational pressure and natural selection across ecologically divergent lineages. We assembled and annotated the complete chloroplast genome of *Paphiopedilum armeniacum* and integrated it with 47 publicly available genomes, representing all four genera of Cypripedioideae. Comparative analyses included genome structure relative synonymous codon usage (RSCU), effective number of codons (ENC) vs. GC3s plots, neutrality analysis, parity rule 2 (PR2) bias analysis, simple sequence repeat (SSR) profiling, and selection pressure assessment (Ka/Ks). Phylogenetic reconstruction was performed using maximum likelihood based on 81 concatenated protein-coding genes. Cypripedioideae chloroplast genomes displayed substantial size variation (147–230 kb) and lineage-specific structural features, including inverted repeat (IR) expansion and small single-copy (SSC) contraction in *Paphiopedilum*. Codon usage was strongly biased toward A/U-ending codons, with minimal influence from mutational pressure. ENC-GC3s plots, neutrality analysis (regression slopes = 0.003–0.324), and PR2 plots consistently indicated that natural selection accounted for >90% of CUB variation across most species. Ka/Ks analysis revealed predominant purifying selection (94.7% of genes with Ka/Ks < 1), with photosystem and ribosomal protein genes under the strongest constraints. Phylogenomic analysis resolved genus-level relationships and showed correlations between genome architecture, codon usage patterns, and ecological distributions. Our findings demonstrate that natural selection, rather than mutational pressure, is the primary driver of codon usage optimization in Cypripedioideae chloroplast genomes. Lineage-specific structural variations—including IR/SSC boundary dynamics, genome size expansion, and *ndh* gene loss—are closely associated with ecological adaptations to temperate, subtropical, and tropical environments. This study provides novel insights into the interplay between selection-driven codon usage, plastome structural evolution, and ecological diversification, offering a genomic framework for understanding adaptive evolution in orchids and other plant lineages.

## 1 Introduction

The orchid family (Orchidaceae) comprises approximately 736 genera and 28,000-30,000 species, representing one of the most species-rich and morphologically diverse groups of flowering plants. This diversity reflects millions of years of adaptation to fluctuating climates and heterogeneous geographic environments, resulting in pronounced phenotypic plasticity (Case et al., 1998; Feng et al., 2024). Within this family, the subfamily Cypripedioideae represents one of five recognized subfamilies (Cox et al., 1997; Li et al., 2011; Zhang et al., 2022). Although species diversity in Cypripedioideae is moderate (~200 extant species), its wide ecological distribution across temperate, subtropical, and tropical regions makes it an ideal model for studying adaptive evolution across environmental gradients. The subfamily encompasses five genera: *Cypripedium*, *Selenipedium*, *Phragmipedium*, *Mexipedium*, and *Paphiopedilum* (Cox et al., 1997; Pemberton, 2013). These genera exhibit distinct biogeographical patterns: *Cypripedium* occurs primarily in temperate to boreal Eurasia and North America, representing the sole temperate-adapted lineage; *Selenipedium*, *Phragmipedium*, and *Mexipedium* are restricted to the Neotropics, ranging from southern Mexico through Central and South America, with *Mexipedium xerophyticum* as a monotypic endemic of Oaxaca, Mexico; while *Paphiopedilum* is concentrated in karst tropical and subtropical regions, spanning the Indian subcontinent, Southeast Asia, the Philippines, and extending southward across the Malay Archipelago to the Solomon and Bismarck Islands (Praptosuwiryo et al., 2024; van der Ent et al., 2015).

Plant species occupying distinct ecological niches frequently exhibit substantial functional and morphological differentiation driven by habitat-specific selective pressures (Shuai et al., 2018; Wainwright, 2007). For instance, xerophytic *Dendrobium* species display succulent leaves, thick cuticles, enlarged epidermal cells, and sunken stomata, reflecting adaptation to karstic, water-limited environments (Liu et al., 2021). By contrast, mesophytic *Dendrobium* species have larger stomata, loosely arranged mesophyll, and thinner cuticles, consistent with life in humid or seasonally variable habitats. These examples highlight how structural and physiological traits are finely tuned to ecological conditions. Despite growing attention to physiological adaptations, the molecular mechanisms underlying these traits remain incompletely understood.

Chloroplast genomes provide a powerful framework for investigating adaptive evolution due to their key functional role in photosynthesis and their unique inheritance patterns (Korpelainen, 2004; Xiong et al., 2021). The uniparental inheritance and low recombination rates of chloroplast DNA preserve phylogenetic signals, facilitating reconstruction of evolutionary histories and population dynamics (Freeland et al., 2020). Although plastid genomes evolve more slowly than nuclear genomes, mutations in non-coding regions and specific coding sequences (CDS) can resolve species- and population-level differentiation (Necsulea and Kaessmann, 2014; Zamai, 2020). Importantly, chloroplast-encoded genes are central to photosynthesis, so genetic variation in these loci can directly reflect selection pressures related to environmental performance. For example, polymorphisms in *psbA* (encoding the PSII D1 subunit) are associated with high-light tolerance, whereas variation in *rbcL* (encoding the Rubisco large subunit) influences carbon fixation efficiency (Chotewutmontri and Barkan, 2020; Lou et al., 2018; Mulo et al., 2009). Comparative studies indicate that plastome evolution is closely linked to ecological adaptation across lineages, including Cypripedioideae (Liu et al., 2022). Although Cypripedioideae plastomes retain the canonical quadripartite structure, lineage-specific structural modifications, such as inverted repeat (IR) expansions or small single-copy (SSC) contractions, large inversions, and repeat accumulation, generate substantial variation in genome size (Guo et al., 2021b; Zhang et al., 2022). In derived lineages, genes including *infA*, *rpl20*, and members of the *ndh* family exhibit positive selection, loss, or pseudogenization, potentially reflecting ecological adaptation (Scobeyeva et al., 2021). Notably, the functional loss of *ndh* genes has been hypothesized to correlate with adaptation to low-light conditions typical of epiphytic or forest-floor environments (Ruhlman et al., 2015). Such patterns underscore the role of plastome evolution in adaptive differentiation and motivate investigation of mechanisms like codon usage bias (CUB), which can influence translational efficiency and adaptive potential (Li and Wei, 2022; Yang et al., 2023).

Codon usage bias (CUB), the non-random utilization of synonymous codons, affects mRNA stability, translation efficiency and accuracy, and protein folding, thereby influencing organismal adaptation and evolutionary trajectories (Behura and Severson, 2013; Parvathy et al., 2022). CUB arises from multiple forces, including mutational pressure, genetic drift, and natural selection, or a combination thereof (Lynch et al., 2016; Shah and Gilchrist, 2011). Comparative analyses of CUB across species reveal systematic differences at gene, genome, and lineage levels, reflecting both evolutionary history and ecological specialization (Zhang et al., 2014). Consequently, codon usage analysis is a robust tool for exploring molecular evolution, gene function optimization, and adaptive evolution at the genomic scale (Parvathy et al., 2022). Despite extensive study of plant CUB, the relationship between CUB and ecological factors in orchids, particularly within the ecologically diverse Cypripedioideae, remains largely unexplored. To address this gap, we conducted a comprehensive analysis of chloroplast genomes from 48 Cypripedioideae species representing all major lineages. Although only one plastome (*Paphiopedilum armeniacum*) was newly sequenced in this study, integration with 47 publicly available genomes enabled the most extensive comparative CUB analysis for this subfamily to date. This dataset spans all four extant genera and encompasses diverse ecological niches from alpine to tropical habitats, allowing the first systematic evaluation of codon usage patterns and their ecological correlates within Cypripedioideae. Our analyses focused on elucidating the evolutionary forces shaping codon usage. Relative synonymous codon usage (RSCU), correspondence analysis (COA), effective number of codons (ENC) versus GC3 (ENC-GC3), parity rule 2 (PR2) plots, and neutrality analyses were applied to quantify codon usage patterns and assess the contributions of mutational bias versus selection. Additionally, gene-level Ka/Ks ratios were calculated to identify selective pressures and explore potential associations with CUB. Specifically, our study aimed to (1) systematically characterize structural variation and CUB patterns across Cypripedioideae chloroplast genomes; (2) evaluate the relative contributions of mutational pressure and natural selection in shaping codon usage bias, thereby identifying the primary evolutionary drivers; and (3) investigate correlations between codon usage in environmentally responsive genes and ecological variables, while comparing genome structural variation among species occupying divergent habitats to elucidate the molecular mechanisms underlying adaptive evolution. We believe that by integrating comparative genomics with codon usage and ecological data, this study provides novel insights into how selection and structural genome evolution jointly shape adaptive differentiation in Cypripedioideae. The findings contribute to a broader understanding of plant adaptive evolution, highlighting mechanisms that may be relevant for conservation, functional genomics, and sustainable utilization of orchids and other plant lineages.

## 2 Materials and methods

### 2.1 Plant material sampling and DNA extraction

Fresh leaf tissue of *P. armeniacum* was collected from Xingyi City, Qianxinan Buyi and Miao Autonomous Prefecture, Guizhou Province, China (25°00′29″N, 104°58′34″E). Voucher specimens were deposited at the Beijing Forestry University (voucher number: CS675-004R0003). Healthy, young leaves were immediately stored in silica gel to preserve DNA integrity and subsequently transported to the laboratory for DNA extraction. All collection procedures were conducted following institutional and national guidelines and complied with local biodiversity conservation regulations. Total genomic DNA was extracted from approximately 100 mg of silica-dried leaf tissue using a modified cetyltrimethylammonium bromide (CTAB) protocol (Liu et al., 2025; Narzary et al., 2015). Genomic DNA was fragmented to 350–400 bp using enzymatic shearing with the NEBNext dsDNA Fragmentase (New England Biolabs, Ipswich, MA, USA). DNA quality and concentration were assessed using a NanoDrop 2000 spectrophotometer (Thermo Fisher Scientific, Waltham, MA, USA) and agarose gel electrophoresis (Schiebelhut et al., 2017). High-quality DNA samples with A260/A280 ratios between 1.8 and 2.0 were used for subsequent library preparation (Abdel-Latif and Osman, 2017).

### 2.2 Genome sequencing and chloroplast genome assembly

Whole genome sequencing libraries were constructed using the Illumina TruSeq DNA PCR-Free Library Preparation Kit following the manufacturer’s protocol. Briefly, approximately 1 μg of genomic DNA was enzymatically fragmented, end-repaired, A-tailed, and ligated to indexed adapters. Size selection was performed using AMPure XP beads (Beckman Coulter, Brea, CA, USA). Library quality and concentration were assessed with an Agilent 2100 Bioanalyzer (Agilent Technologies, Santa Clara, CA, USA) and quantified by qPCR (Brena et al., 2006). Qualified libraries were sequenced on an Illumina NovaSeq 6000 platform (Illumina, San Diego, CA, USA) using 150 bp paired-end sequencing chemistry, generating approximately 4.5 Gbp of raw data (Foox et al., 2021; Modi et al., 2021).

Raw reads were quality-checked using FastQC v0.20.1 (Andrews, 2012) to remove adapter sequences, low-quality bases (Q < 20), and reads shorter than 50 bp. Chloroplast genome assembly was conducted using GetOrganelle v1.7.4.1 (Jin et al., 2020) and NOVOPlasty v4.3.4 (Dierckxsens et al., 2017). Approximately 15 million paired-end reads were used for assembly. Parameters were set as k-mer lengths of 21, 45, 65, 85, and 105 (-k 21,45,65,85,105), 15 extension rounds (-R 15), 100 threads (-t 100), and the embryophyta plastid database (-F embplant_pt) as reference. Seed reads were initially mapped to the reference using Bowtie2 v2.4.1, followed by iterative extension. *De novo* assembly utilized SPAdes v3.15.5 (Zaki, 2001) integrated in GetOrganelle. The observed insert size was 237.2 bp ± 66.3 bp, with an average chloroplast coverage of 360.5×.

### 2.3 Genome annotation and visualization

Annotation of the assembled plastome was performed using GeSeq (https://chlorobox.mpimp-golm.mpg.de/geseq.html) (Tillich et al., 2017) and CPGAVAS2 (http://47.96.249.172:16019/analyzer/home) (Shi et al., 2019), with reference plastomes from closely related orchids. Protein-coding sequences (CDS), ribosomal RNA (rRNA), and transfer RNA (tRNA) genes were identified and annotated. tRNA genes were further validated with tRNAscan-SE v2.0 (Chan et al., 2021). Gene boundaries and intron-exon structures were manually curated by comparison with reference genomes and BLASTp alignments (E-value < 1e-5) against the NCBI non-redundant protein database. LSC, SSC, and IR regions were identified by aligning IR boundaries. Circular genome maps were generated using CPGView (http://www.herbalgenomics.org/cpgview/) (Liu et al., 2023) to display gene organization, GC content, and repeat elements. The complete *P. armeniacum* plastome was deposited in GenBank (PX501760) on 18-Nov-2025.

### 2.4 Chloroplast genome data acquisition and quality control

Complete chloroplast genomes of Cypripedioideae species were obtained from NCBI GenBank (https://www.ncbi.nlm.nih.gov/genbank/). After filtering for completeness (>95%), annotation quality, and removing duplicates or misidentified sequences, 47 genomes were retained. Combined with our newly sequenced *P. armeniacum*, the dataset included 48 species comprising four genera: *Cypripedium* (11), *Paphiopedilum* (30), *Phragmipedium* (6), and *Mexipedium* (1). All sequences were stored in FASTA format with accompanying GenBank annotations. Quality control included manual verification in BioEdit v7.2.6, BLAST alignment checks, and extraction of CDSs using custom Perl scripts. Only complete CDS without internal stop codons and divisible by three were retained for codon usage analyses. Multiple sequence alignment was performed using MAFFT v7.475 (Katoh and Standley, 2013).

### 2.5 Relative synonymous codon usage analysis

RSCU quantifies codon usage bias as the ratio of observed codon frequency to expected frequency under the assumption of equal synonymous codon usage (Sharp et al., 1986). Values > 1 indicate positive bias, <1 indicate negative bias, and =1 indicate no bias. RSCU was computed for all species using custom Python scripts with Biopython v1.79. Stop codons and genes with nonstandard nucleotides were excluded. Heatmaps were generated using the pheatmap package v1.0.12 in R v4.2.2, with hierarchical clustering to visualize codon usage relationships among species.

### 2.6 Neutrality analysis

Neutrality analysis distinguishes the contributions of mutational pressure versus natural selection on codon usage (Sueoka, 1995). GC content at the first and second codon positions (GC12) and at the third codon position (GC3) were calculated for each gene. GC3 is more sensitive to mutational pressure, whereas GC12 is under stronger functional constraints. Linear regression analysis was employed to examine the correlation between GC12 and GC3, a slope approaching 1 suggests mutation-driven evolution, whereas a slope near 0 indicates the dominance of natural selection. The regression coefficient (R²) quantifies correlation strength (Sueoka, 1995). Analyses were performed using custom Python scripts, with ggplot2 v3.4.0 for high-resolution scatter plots, color-coded by genus.

### 2.7 ENC-GC3 analysis

The effective number of codons (ENC) measures codon usage bias (Wright, 1990), and ranges from 20 (extreme bias) to 61 (no bias). ENC-GC3s plots compare observed ENC values to expectations based on GC3s content indicating selection versus mutation dominance. Values near the curve indicate mutation-driven bias; deviations suggest selection. ENC and GC3 values were calculated with CodonW v1.4.4. Scatter plots were visualized with ggplot2 in R, with point sizes size proportional to ENC and colors representing species. Genus-level faceting facilitated comparative evolutionary insights.

### 2.8 Simple sequence repeat analysis

SSRs, or microsatellites, are tandem repeats valuable for population genetics and marker development (Kashi and King, 2006). SSRs in 48 Cypripedioideae plastomes were identified using MISA v2.1 (Beier et al., 2017) and custom Python scripts. Parameters were: ≥10 mononucleotide, ≥6 dinucleotide, ≥5 tri- to pentanucleotide, and ≥3 hex- to decanucleotide repeats. Only perfect SSRs were considered. SSRs were classified by repeat unit length, genomic location (CDS, intron, intergenic), and repeat number. Visualizations were performed using R scripts.

### 2.9 Ka/Ks analysis

Selection pressures on chloroplast CDSs were evaluated by calculating Ka/Ks ratios. Orthologous gene across 48 species (41,790 comparisons) were aligned with MAFFT v7.475 and trimmed using TrimAl v1.4.rev15 (-automated1). Alignments were converted to Axt format for Ka/Ks calculation using KaKs_Calculator v2.0 (NG method). Significance was assessed via one-sample *t*-tests with Bonferroni correction. Genes were categorized as strong purifying (Ka/Ks < 0.5), relaxed purifying (0.5-1), or positively selected (Ka/Ks > 1). Functional categories followed CPGView: photosystems I/II, ATP synthase, cytochrome b_6_f, NADH dehydrogenase, Rubisco, RNA polymerase, ribosomal proteins, and hypothetical reading frames. Pairwise comparisons were evaluated with Wilcoxon rank-sum tests, effect sizes by Cohen’s d, and Ka/Ks correlations assessed via Pearson correlation. Outliers (Ka/Ks > 10) were excluded.

### 2.10 Chloroplast genome collinearity and structural analysis

Collinearity was analyzed using R v4.2.2 with genoPlotR, ComplexHeatmap, circlize, and grid packages (Guy et al., 2010). Gene tables included gene name, location, orientation, and functional classification, with color-coding by function. Pairwise BLASTN alignments (e < 1e-5) determined homology and synteny. DNA segments and comparisons were visualized with dna_seg and comparison functions in R. Structural analysis included genome size, GC content, gene numbers, regional LSC/SSC/IR boundaries, and gene composition (protein-coding, tRNA, rRNA). GC content at codon positions (GC1, GC2, GC3) was examined to infer selection.

### 2.11 Phylogenetic analysis of chloroplast genomes

Phylogenetic reconstruction was performed based on 110 Orchidaceae chloroplast genomes, comprising 102 Cypripedioideae species representing all four extant genera and 8 outgroup species from Apostasioideae and Vanilloideae, representing early-diverging subfamilies within Orchidaceae. Protein-coding genes were extracted from annotated chloroplast genomes using custom Python scripts and individually aligned using MAFFT v7.505 (Katoh and Standley, 2013) with the L-INS-i algorithm for accurate alignment of sequences with conserved domains. Ambiguously aligned regions were identified and removed using trimAl v1.4.rev15 (Capella-Gutiérrez et al., 2009) with the -automated1 option, which automatically selects the optimal trimming parameters based on alignment characteristics. After quality assessment, 81 protein-coding genes passing stringent quality thresholds were retained for phylogenetic analysis. Individual gene alignments were concatenated into a supermatrix using FASconCAT-G v1.05 (Kück and Longo, 2014). Maximum likelihood (ML) phylogenetic analysis was conducted using IQ-TREE2 v2.2.0 (Minh et al., 2020). The best-fit substitution model was automatically selected by ModelFinder (Kalyaanamoorthy et al., 2017) using the Bayesian Information Criterion (BIC). Branch support was rigorously assessed using three complementary approaches: (1) the Shimodaira-Hasegawa approximate likelihood ratio test (SH-aLRT) with 1,000 replicates (Guindon et al., 2010); (2) approximate Bayes posterior probability (Anisimova et al., 2011); and (3) ultrafast bootstrap approximation (UFBoot2) with 1,000 replicates (Hoang et al., 2018). Following the recommendations of Minh et al. (2013), nodes with SH-aLRT ≥80% and UFBoot ≥95% were considered strongly supported (Minh et al., 2013). Phylogenetic trees were visualized and annotated using ggtree v3.15.0 (Yu et al., 2017) in R v4.2.3, with branch tips color-coded by genus to facilitate interpretation of taxonomic relationships.

### 2.12 Multi-group statistical comparison analysis

A comparative statistical analysis was conducted to assess variation in chloroplast genome characteristics across the three major genera of Cypripedioideae: *Paphiopedilum*, *Cypripedium*, and *Phragmipedium* (*Mexipedium* was excluded due to its monotypic status). Eight genomic parameters were examined: evolutionary rate (sum of root-to-tip branch lengths), GC content, genome size, gene density, coding ratio, number of tRNA genes, inverted repeat (IR) length, and AT skew. As most data violated assumptions of normality, non-parametric Kruskal-Wallis H tests were used to evaluate overall differences among genera for each parameter, with significance levels set at P < 0.05, P < 0.01, and P < 0.001. Where a significant overall difference was detected, post-hoc pairwise comparisons were performed using Dunn’s test with Bonferroni correction. Inter-relationships among all parameters were explored by calculating Pearson correlation coefficients across the complete dataset; significance was assessed via two-tailed t-tests. Data were visualized using violin plots (showing probability density) with embedded boxplots (showing median and interquartile range) and overlaid individual data points. Correlation results were presented as a heatmap. All analyses and visualizations were performed in R (v4.2.2) using the rstatix, ggpubr, corrplot, ggplot2, and patchwork packages.

## 3 Results

### 3.1 Chloroplast genome assembly, structure and characteristics of Cypripedioideae

**Figure 1:**
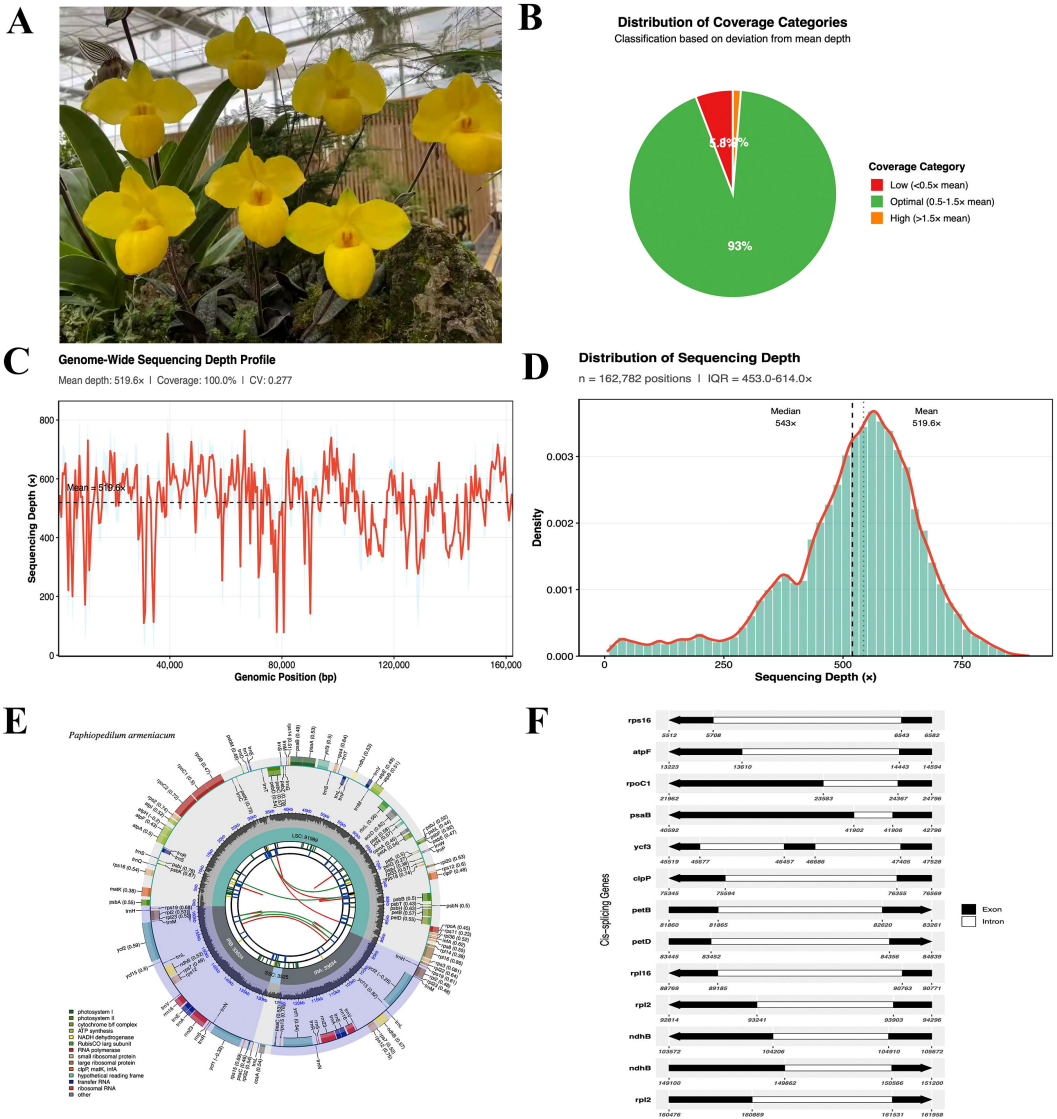
The complete chloroplast genome of *Paphiopedilum armeniacum* and sequencing quality assessment. **(A)** Photograph showing the characteristic yellow flowers of *P. armeniacum*, an endangered orchid species endemic to southwestern China. **(B)** Distribution of coverage categories across the genome. The pie chart displays the proportion of genomic positions classified by sequencing depth relative to mean coverage: optimal coverage (0.5-1.5× mean, green), low coverage (<0.5× mean, red), and high coverage (>1.5× mean, orange). **(C)** Genome-wide sequencing depth profile. The line graph illustrates sequencing depth variation across the entire 162,782 bp chloroplast genome. Red line represents mean depth calculated across 500 bp sliding windows, with gray shading indicating ±1 standard deviation. Black dashed line marks the overall mean depth of 519.6×. The relatively stable coverage pattern demonstrates consistent sequencing quality across all genomic regions. **(D)** Distribution of sequencing depth. The histogram (teal bars) shows the frequency distribution of depth values across all 162,782 genomic positions, overlaid with a kernel density estimate (red curve). **(E)** Circular representation of the *P. armeniacum* chloroplast genome. The outermost circle displays genes on the forward strand (outside) and reverse strand (inside), color-coded by functional category. The second circle shows GC content variation (dark gray, high GC; light gray, low AT). Inner circles illustrate the quadripartite structure comprising large single copy (LSC), small single copy (SSC), and two inverted repeat (IR) regions. Gene positions and orientations are indicated with arrows. Notable features include the characteristic IR expansion and SSC contraction typical of Paphiopedilum species. **(F)** Gene structure diagrams of selected chloroplast genes. Exon-intron organization of representative genes is shown with exons depicted as black boxes and introns as connecting lines.

We successfully assembled the complete chloroplast genome of *P. armeniacum* (**Figure 1A**) using GetOrganelle v1.7.4.1 as the primary assembly tool. From 15 million paired-end reads, a circular plastome of 162,782 bp was obtained with high sequencing quality (mean depth: 519.6×, median: 543×) (**Figures 1C, D**). The assembly achieved 100% genome coverage with uniform read depth across all structural regions (**Figure 1B**). Depth distribution approximated a normal pattern with an interquartile range of 453.0-614.0×(**Figure 1D**), indicating minimal technical bias and confirming suitability for downstream variant detection and comparative analyses.

The *P. armeniacum* plastome exhibits the canonical quadripartite structure typical of Cypripedioideae, consisting of a large single-copy (LSC) region of 91,989 bp, a small single-copy (SSC) region of 4,363 bp, and two inverted repeats (IRa and IRb) of 33,436 bp each (**Figure 1E**). Nucleotide composition analysis revealed an AT-rich genome, with adenine (A) at 31.93%, thymine (T) at 32.68%, cytosine (C) at 17.96%, and guanine (G) at 17.44% (**Supplementary Table S1**). A total of 125 genes were annotated in the *P. armeniacum* chloroplast genome, including 82 protein-coding genes, 37 tRNAs, and 6 rRNAs, spanning functional categories such as photosystems I and II, ATP synthase, cytochrome b_6_f complex, and core genetic system components (**Figure 1**, **Supplementary Table S1**). Notably, the genome displays IR expansion and SSC contraction typical of *Paphiopedilum* species, with genes such as *ycf1* and *psaC* relocated from the SSC to IR regions. The overall GC content (35.4%) and codon-position GC distribution (GC1: 44.83%, GC2: 37.31%, GC3: 30.09%) reflect the AT bias prevalent in slipper orchid plastomes. Exon-intron architecture was conserved across representative genes (**Figure 1F**), consistent with known Orchidaceae plastome organization and providing a robust framework for comparative genomic and phylogenetic analyses.

A comprehensive survey of 48 complete Cypripedioideae chloroplast genomes revealed substantial interspecific variation in genome architecture and nucleotide composition (**Supplementary Table S1**). Genome size ranged from 147,384 bp to 229,932 bp, with a mean of 164,284 bp, representing a variation of ~82.5 kb. This variation markedly exceeds that of most angiosperms, where plastomes are generally highly conserved. Heterogeneity in genome size was primarily driven by differential expansion and contraction of genomic regions, especially the LSC and IR boundaries. Total GC content ranged from 26.66% to 37.82%, with a mean of 34.94%, indicating a moderate AT bias typical of plastid genomes. Notably, several *Cypripedium* species exhibited lower GC content relative to the more conserved ~37.6% commonly observed in terrestrial angiosperm plastomes.

Among the 11 *Cypripedium* species, genome sizes varied from 157,050 bp (*C. macranthos*) to 229,932 bp (*C. micranthum*), averaging 188,297 bp (**Supplementary Table S1**). Expansion was primarily due to proliferation of AT-rich repeat sequences in noncoding regions, particularly within the LSC. GC content ranged from 26.66% to 37.82% (mean 32.01%), substantially lower than that of *Paphiopedilum* and *Phragmipedium*. AT enrichment was especially pronounced in expanded intergenic and tandem repeat regions. Large plastomes, such as those of *C. subtropicum* (212,668 bp) and *C. tibeticum* (197,815 bp), represent the largest chloroplast genomes reported in Orchidaceae, with size expansion strongly correlated with accumulation of SSRs and AT-rich tandem repeats in the LSC. Positional GC content was relatively uniform (GC1: 45.18%, GC2: 37.77%, GC3: 30.41%), while GC3s averaged 27.40%, reflecting a strong bias for AT-ending codons, likely associated with adaptation to temperate and alpine environments. Several *Cypripedium* species also exhibit a 75–90 kb inversion spanning *trnG-UCC* to *trnP-UGG* within the LSC, a structural feature distinguishing certain sections of the genus and suggesting independent rearrangement events during diversification.

In contrast, the chloroplast genomes of 30 *Paphiopedilum* species were smaller and structurally distinct, ranging from 154,569 bp to 163,243 bp, with a mean of 159,016 bp (**Supplementary Table S2**). They are characterized by pronounced IR expansion and dramatic SSC contraction, a pattern unusual in autotrophic angiosperms but common in this genus. Total GC content was relatively uniform (35.29–36.34%, mean 35.70%), ~3.7 percentage points higher than in *Cypripedium*, reflecting reduced AT-rich noncoding sequence content and compact genome architecture. Nucleotide composition averaged 31.68% A, 32.61% T, 18.05% C, and 17.65% G, with positional GC distribution of GC1: 45.02%, GC2: 37.18%, GC3: 29.87%, and GC3s: 26.91%. The most striking structural feature is extensive IR expansion into the SSC, reducing it to as little as 524 bp in some species and relocating up to six genes originally in the SSC into the IRs. LSC/IR boundaries remained relatively stable, whereas IR/SSC boundaries showed substantial variation, particularly within subgenus *Parvisepalum*. Notably, multiple *Paphiopedilum* species exhibit complete pseudogenization or loss of several *ndh* genes, indicative of ongoing plastome degradation in this lineage.

The six *Phragmipedium* species analyzed possessed the most compact and structurally uniform plastomes, ranging from 147,384 bp to 151,238 bp, with a mean of 149,927 bp (**Supplementary Table S3**). GC content was highly consistent (36.04-36.50%, mean 36.21%), the highest among the three genera, reflecting a reduced accumulation of AT-rich repeats and efficient genome organization. Nucleotide composition was uniform (31.27% A, 32.52% T, 18.32% C, 17.89% G), and positional GC distribution was tightly clustered (GC1: 45.71%, GC2: 37.88%, GC3: 30.56%, GC3s: 27.67%). The IR/SSC boundaries in *Phragmipedium* exhibited moderate IR expansion and SSC contraction, distinct from the patterns in *Cypripedium* and *Paphiopedilum*. The relatively stable genome structure and high GC content may reflect selective pressures associated with its Neotropical distribution and ecological specialization. Phylogenetic analyses consistently placed *Phragmipedium* as sister to *Mexipedium*, forming a clade distinct from *Cypripedium* and *Paphiopedilum* lineages.

### 3.2 RSCU analysis

**Figure 2:**
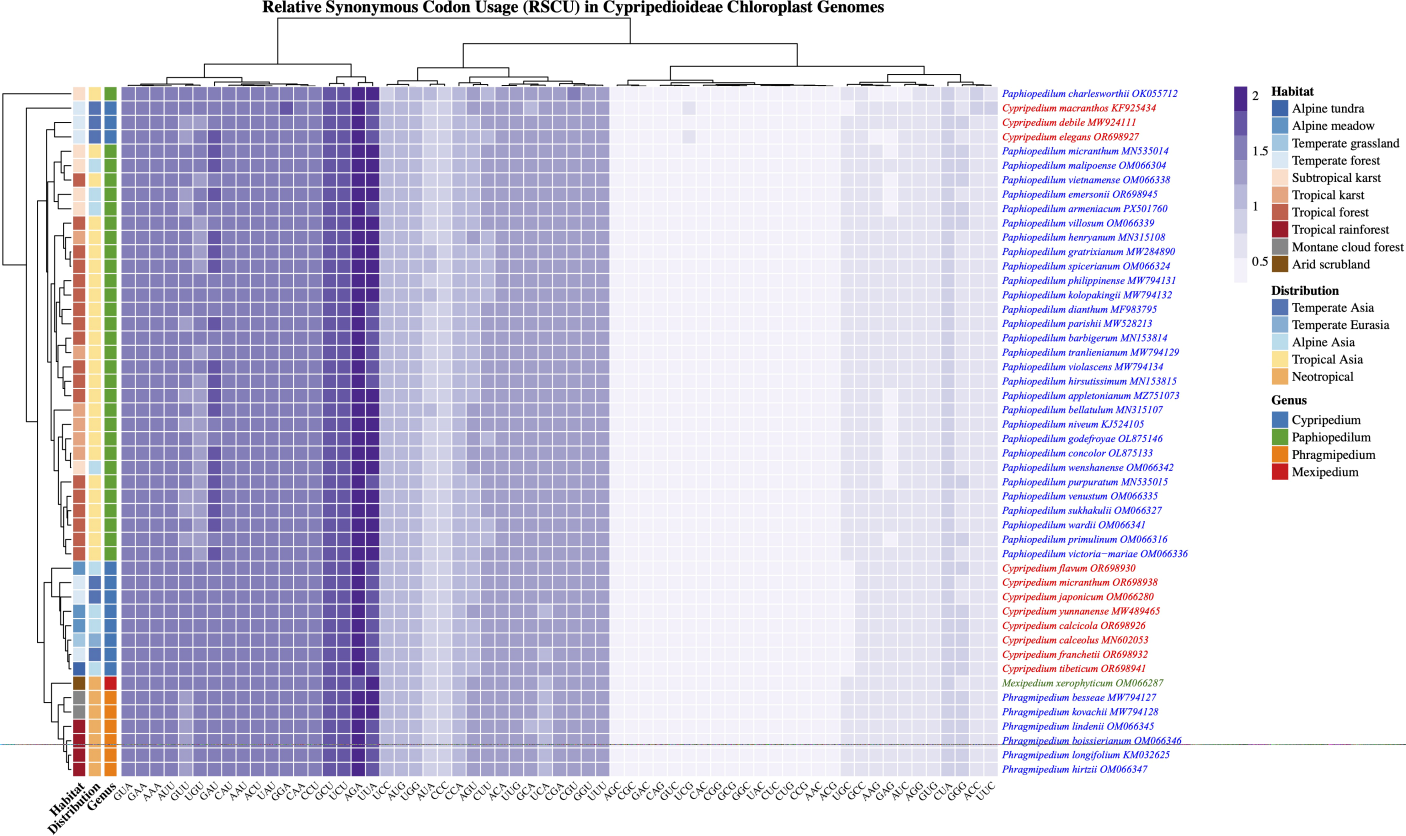
Heatmap visualization of relative synonymous codon usage (RSCU) patterns across 48 Cypripedioideae species chloroplast genomes. Each row represents a different species from subfamily Cypripedioideae with GenBank accession numbers, encompassing four genera: *Cypripedium*, *Paphiopedilum*, *Phragmipedium*, and *Mexipedium*. Each column represents a codon with its corresponding amino acid in parentheses. RSCU values are color-coded with lighter colors indicating lower values (≤0.5) and deeper colors indicating higher values (>3.0).

We analyzed RSCU across the complete chloroplast genomes of 48 Cypripedioideae species, comprising 11 *Cypripedium*, 30 *Paphiopedilum*, 6 *Phragmipedium*, and 1 *Mexipedium* (**Supplementary Table S2A**; **Figure 2**). A consistent CUB was observed across the subfamily, characterized by a pronounced preference for codons ending in A or T at the third codon position, whereas G- or C-ending codons were underrepresented. This AT-rich bias was evident across all degenerate amino acids, indicating non-random synonymous codon selection in slipper orchid plastomes. For example, leucine codons UUA and UUG were the most frequently used, with mean RSCU values of 1.863 and 1.270, respectively, while CUA, CUC, CUG, and CUU showed markedly lower usage. Similarly, arginine exhibited strong preference for AGA over CGA, CGC, CGG, and CGU. These patterns reflect the overall high AT content observed in Cypripedioideae chloroplast genomes.

Codon usage bias was highly conserved across genera. *Cypripedium* exhibited a strong AT-rich bias with minimal interspecific variation. *Paphiopedilum*, despite its higher species richness and broader geographic distribution, displayed a similar codon bias profile, as did *Phragmipedium* and *Mexipedium xerophyticum*. A Kruskal-Wallis test detected no significant differences among genera, suggesting that AT-biased codon usage was established prior to the diversification of major Cypripedioideae lineages. Hierarchical clustering based on RSCU values largely grouped species by genus. *Cypripedium* formed a distinct cluster, separate from a combined *Paphiopedilum-Phragmipedium* group. Several codons exhibited extreme bias: UUA, AAA, AGA, and UUU were consistently highly preferred, whereas 16 GC-rich codons, including CGC, CGG, CUC, and GAC, were strongly avoided. This pronounced divergence highlights strong directional selection favoring AT-rich synonyms in Cypripedioideae chloroplast genomes, consistent with patterns observed in other plant lineages experiencing diverse ecological pressures.

### 3.3 SSR analysis

**Figure 3:**
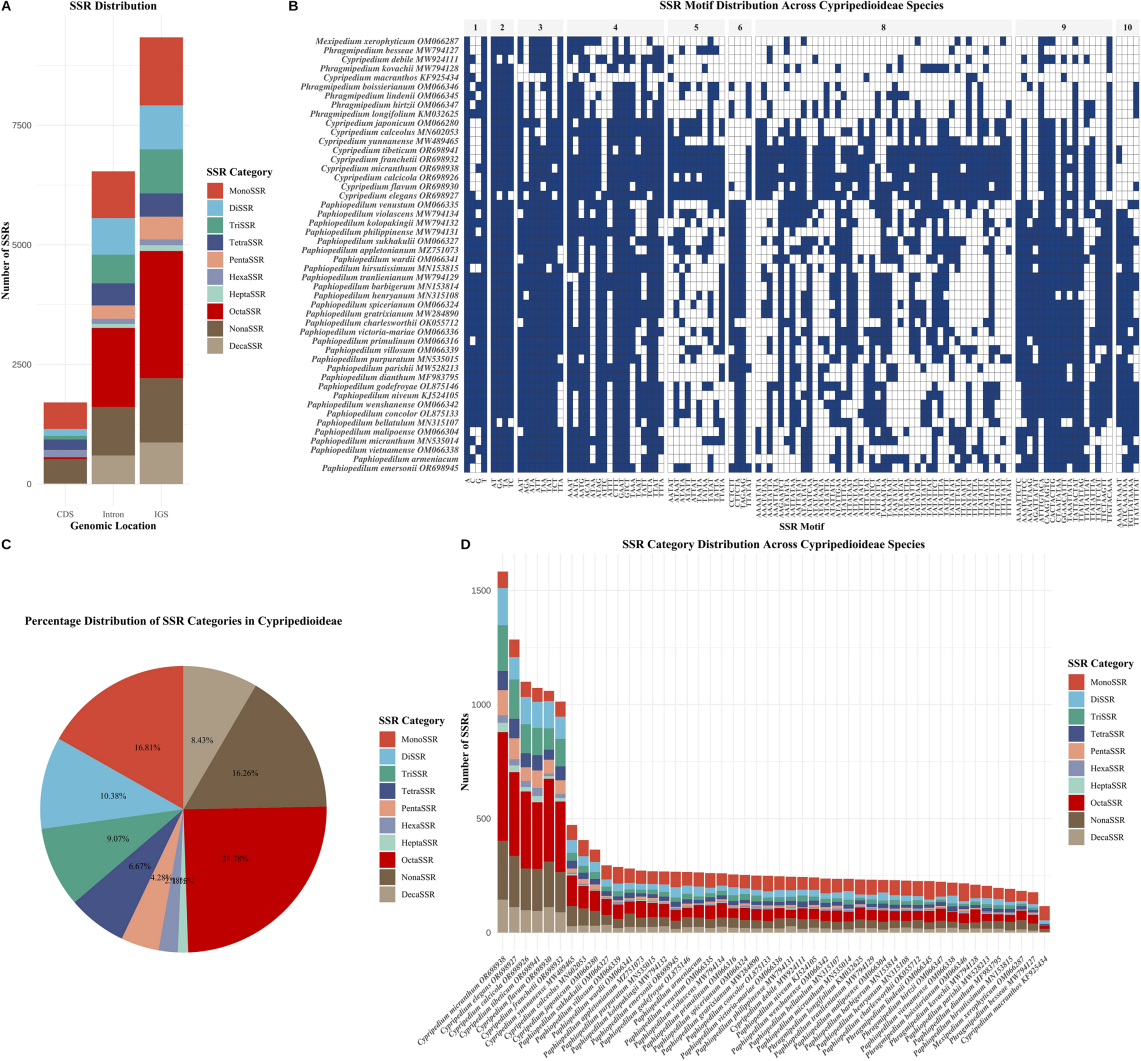
Comprehensive analysis of simple sequence repeats (SSRs) in chloroplast genomes of 48 Cypripedioideae species. **(A)** Distribution of SSR categories across different genomic locations including coding sequences (CDS), intergenic spacers (IGS), and other genomic regions. Bar heights represent the count of each SSR category with different colors indicating MonoSSR through ExtendedSSR types. **(B)** Heatmap displaying presence/absence patterns of SSR motifs across all analyzed Cypripedioideae species. Blue cells indicate presence (1) and white cells indicate absence (0) of specific SSR motifs. Motifs are organized by repeat unit length (1-10+ bp) and grouped in panels above the heatmap. Species are clustered based on SSR motif similarity using Ward’s hierarchical clustering method. **(C)** Pie chart showing the percentage distribution of SSR categories identified across all species. Percentages are calculated based on total SSR count and represent the relative abundance of each category. **(D)** Stacked bar chart illustrating the distribution and abundance of SSR categories across individual Cypripedioideae species. Each bar represents one species with colors corresponding to different SSR categories. Species are ordered by total SSR count for better visualization of patterns.

SSR analysis identified 17,584 perfect microsatellite loci across the 48 Cypripedioideae chloroplast genomes, with individual species containing 78–293 SSRs per genome (**Figure 3D**; **Supplementary Table S3D**). This substantial interspecific variation in SSR abundance correlated with differences in genome size and structural organization among the four genera. The distribution of SSR categories revealed an unusual pattern: octanucleotide repeats (OctaSSR) were the most abundant at 24.78% (4,358 loci), followed by mononucleotide repeats (MonoSSR, 16.81%, 2,955 loci) and nonanucleotide repeats (NonaSSR, 16.26%, 2,860 loci) (**Figure 3C**; **Supplementary Table S3C**). Decanucleotide repeats (DecaSSR) accounted for 8.43% (1,482 loci), while shorter repeat units, namely dinucleotide (DiSSR, 10.38%), trinucleotide (TriSSR, 9.07%), tetranucleotide (TetraSSR, 6.67%), pentanucleotide (PentaSSR, 4.28%), hexanucleotide (HexaSSR, 2.18%), and heptanucleotide (HeptaSSR, 1.15%), represented progressively smaller proportions. The predominance of longer repeat units contrasts with most angiosperm chloroplast genomes, where mono- and dinucleotide repeats typically dominate, suggesting distinctive mutational dynamics in Cypripedioideae plastomes.

Analysis of genomic compartment distribution revealed pronounced location-specific enrichment (**Figure 3A**; **Supplementary Table S3A**). Intergenic spacer (IGS) regions harbored 9,279 SSRs (52.76% of total), with octanucleotide (2,661 loci), nonanucleotide (1,343 loci), and mononucleotide (1,423 loci) repeats being the most abundant. Intronic regions contained 5,538 SSRs (31.49%) with a similar categorical distribution, whereas CDSs contained only 1,707 SSRs (9.71%), showing markedly reduced frequencies across all categories. This pronounced IGS enrichment aligns with the expectation that noncoding regions experience relaxed purifying selection relative to CDS, permitting accumulation of repetitive elements without deleterious functional consequences. SSR motif composition revealed a strong nucleotide bias consistent with the AT-rich nature of Cypripedioideae chloroplast genomes (**Figure 3B**; **Supplementary Table S3B**). Among mononucleotide repeats, A and T homopolymers predominated over G and C tracts across all species, consistent with the subfamily’s mean GC content of 31-36%. The most ubiquitous SSR motifs, which are present in ≥90% of species, included A and T mononucleotide repeats, AT and TA dinucleotide repeats, and several trinucleotide motifs (AAT, ATA, ATT, TAA, TAT, TTA), suggesting these elements have been maintained since the early diversification of Cypripedioideae.

Hierarchical clustering based on SSR presence/absence patterns largely recapitulated known phylogenetic relationships, with congeneric species clustering together, although some exceptions indicated homoplasy in SSR gain/loss events. Species-specific SSR profiles exhibited considerable heterogeneity in total counts and repeat types (**Figure 3D**; **Supplementary Table S3D**), with *Paphiopedilum* species generally exhibiting higher SSR densities than *Cypripedium*, potentially reflecting the differential IR boundary dynamics and compact genome architecture characteristic of tropical lineages. The pronounced enrichment of longer repeat units (octa- through decanucleotide repeats) represents an unusual evolutionary pattern, suggesting involvement of replication slippage, recombination-mediated processes, or mobile element activity. The strong association between SSR distribution and genomic compartment (IGS > intron > CDS) reflects hierarchical selective constraints across functional domains. Notably, highly polymorphic SSR loci in hypervariable IGS regions, particularly those flanking genes such as *rps16-trnQ*, *ycf1*, and *trnH-psbA*, represent promising candidates for DNA barcoding and fine-scale phylogeographic studies aimed at resolving species boundaries in this taxonomically complex subfamily.

### 3.4 ENC-GC3 analysis

The ENC quantifies the extent of synonymous codon usage bias, ranging from 20, indicating extreme bias with only one codon used per amino acid, to 61, reflecting uniform usage of all synonymous codons. To evaluate the relative influence of mutational pressure versus natural selection on codon usage patterns, we conducted ENC-GC3s analysis. This method compares observed ENC values against theoretical expectations based on GC content at synonymous third codon positions (GC3s) using Wright’s formula: ENC = 2 + GC3s + 29/[GC3s² + (1 − GC3s)²]. Genes positioned on or near the theoretical curve are primarily influenced by compositional constraints, whereas genes lying substantially below the curve suggest additional selective forces shaping synonymous codon choice.

**Figure 4:**
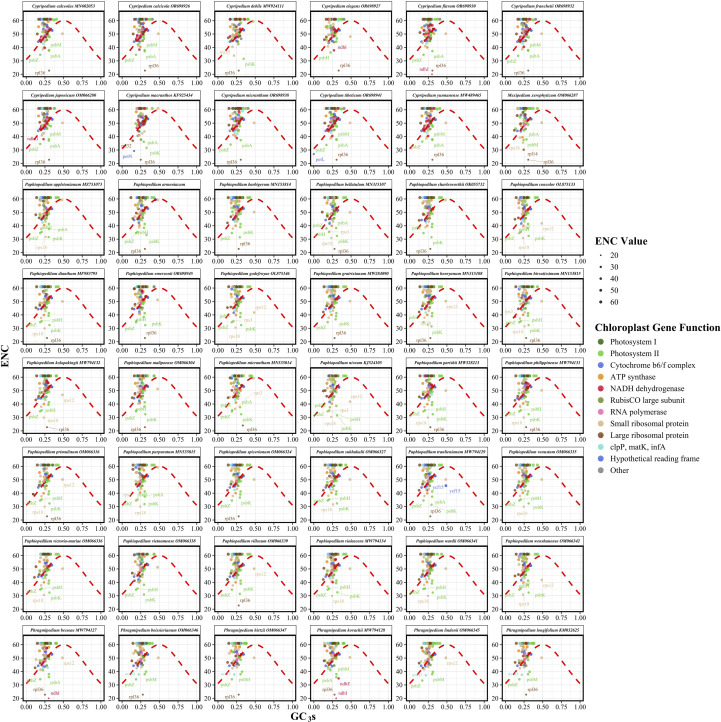
Effective number of codons (ENC) plotted against GC content at synonymous third codon positions (GC3s) for 48 Cypripedioideae species. Each panel displays species-specific codon usage patterns with individual genes represented as colored points scaled proportionally to ENC values. The theoretical ENC curve (red dashed line) represents the expected relationship under neutral evolution with compositional constraints as the sole evolutionary force.

Analysis of 48 Cypripedioideae species spanning four genera, namely *Cypripedium*, *Paphiopedilum*, *Phragmipedium*, and *Mexipedium*, revealed moderate codon usage bias across chloroplast protein-coding genes (**Figure 4**; **Supplementary Table S4A**). Mean ENC values ranged from 51.11 in *Paphiopedilum dianthum* to 53.02 in *Phragmipedium lindenii*, with a subfamily average of 52.09 and low interspecific variation. GC3 content exhibited limited divergence, ranging from 0.2539 in *Paphiopedilum charlesworthii* to 0.2656 in *Cypripedium macranthos*, averaging 0.2583 across the subfamily. The number of analyzed genes per species varied from 72 to 88, reflecting differences in gene complement completeness. Representative species such as *P. armeniacum* showed values consistent with genus-level trends.

At the gene level, both ENC and GC3s varied substantially, with most genes distributed below Wright’s theoretical curve (**Figure 4**). This systematic deviation indicates that natural selection, rather than mutational pressure alone, is the principal force shaping synonymous codon usage in Cypripedioideae plastomes. The consistent downward deviation implies selective pressures related to translational efficiency, protein folding, and functional optimization.

Among genera, *Cypripedium* species exhibited slightly higher mean ENC values than *Paphiopedilum*, suggesting subtle genus-level differences in codon usage constraint. Codon usage bias also differed across functional gene categories (**Supplementary Table S4B**). Photosystem I genes displayed the weakest bias, indicative of relatively balanced synonymous codon usage. In contrast, the RubisCO large subunit (*rbcL*) showed the strongest bias, consistent with intense purifying selection on this critical carbon-fixation enzyme. Photosystem II genes exhibited intermediate bias, while RNA polymerase subunits displayed relatively uniform patterns. NADH dehydrogenase genes, analyzed in a subset of species due to gene loss in some lineages, showed high variability, potentially reflecting relaxed selection following functional deterioration.

GC3 content similarly varied among functional categories. Hypothetical chloroplast reading frames (*ycf* genes) had the highest mean GC3s, whereas cytochrome b_6_f complex genes had the lowest, with several approaching zero. The *rbcL* gene displayed highly constrained GC3s, aligning with its conserved codon usage. Small ribosomal protein genes showed the broadest GC3s variation, highlighting diverse evolutionary pressures across ribosomal components.

Overall, the consistent distribution of genes below the theoretical ENC-GC3 curve across all functional categories confirms the predominant role of natural selection in maintaining codon usage patterns in Cypripedioideae chloroplast genomes. While mutational bias establishes baseline GC3 content, selective forces associated with translational efficiency, protein stability, and expression optimization exert stronger influence. Essential photosynthetic genes, particularly *rbcL* and core photosystem subunits, exhibited the greatest deviation from neutrality, underscoring the action of translational selection on highly expressed, functionally critical genes. These observations are consistent with patterns reported in other angiosperm chloroplast genomes, where photosynthetic genes typically exhibit stronger codon usage bias than housekeeping or structural genes.

### 3.5 Neutrality analysis

**Figure 5:**
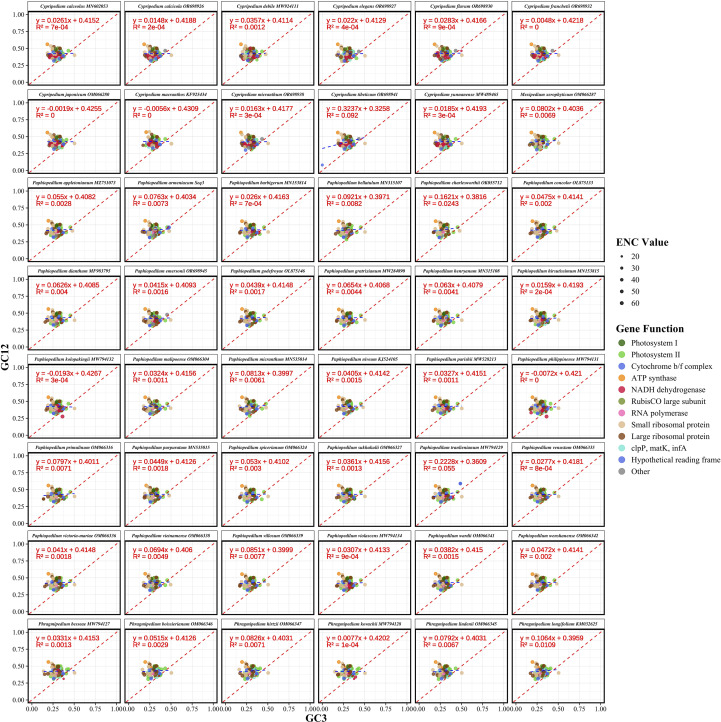
Neutrality analysis of codon usage patterns in Cypripedioideae chloroplast genomes. Gene function colored analysis showing the relationship between GC content at the third codon position (GC3) and GC content at the first and second codon positions (GC12) across 48 species from subfamily Cypripedioideae. The blue dashed line represents the linear regression fit, while the red dashed line represents the neutral expectation (Y=X). Each panel represents a different species from the subfamily Cypripedioideae, including representatives from four genera: *Cypripedium*, *Paphiopedilum*, *Phragmipedium*, and *Mexipedium*.

Neutrality analysis was performed to evaluate the relative contributions of mutational pressure and natural selection in shaping codon usage across 48 Cypripedioideae species (**Figure 5**). Linear regression between GC content at the first and second codon positions (GC12) and at the third position (GC3) revealed very weak correlations, with coefficients of determination (R^2^) ranging from 0.0000 to 0.0920 and a mean of 0.0051. Regression slopes varied from -0.0193 to 0.3237, averaging 0.0535, markedly deviating from the neutral expectation of 1.0. These near-zero slopes and minimal correlations indicate that GC12 and GC3 evolved largely independently, providing strong evidence that natural selection, rather than mutational bias, is the dominant force shaping synonymous codon usage in this subfamily.

At the genus level, *Cypripedium* species exhibited slopes from -0.0056 to 0.3237. *C. tibeticum* showed the strongest apparent mutational influence, with a slope of 0.3237 and R^2^ of 0.0920, suggesting that approximately 32% of its codon usage variation may arise from mutation. In contrast, the 30 *Paphiopedilum* species displayed highly consistent patterns, with slopes ranging from -0.0193 to 0.2228 and R^2^ values between 0.0000 and 0.0550, implying that natural selection accounts for 92-95% of codon usage variation. The reference species *P. armeniacum* exhibited a slope of 0.0763 and R² of 0.0073, closely aligning with congeners and confirming that mutational pressure contributes only ~7.6% to codon bias, with selection governing the remainder.

Functional gene category analysis revealed differential selective pressures across chloroplast pathways. Photosystem I and II genes had mean GC3 values of 0.3165 and 0.3179, respectively, higher than translational machinery genes, which averaged 0.2712 for small and 0.2791 for large ribosomal proteins. The RubisCO large subunit (*rbcL*) exhibited the highest mean GC12 (0.5041) and a moderate GC3 (0.3008), reflecting strong purifying selection acting on its functionally critical amino acid sequence. Hypothetical chloroplast reading frames (*ycf* genes) displayed greater compositional variation, consistent with relaxed selective constraints.

Neutrality plots for all 48 species consistently positioned data points below the diagonal line GC12 = GC3, with most clustering in narrow GC12 and GC3 ranges of 0.38-0.44 and 0.26-0.32, respectively. This pattern indicates constrained nucleotide composition across both phylogenetic and ecological diversity. Exceptions, including *C. tibeticum* and *Paphiopedilum tranlienianum*, exhibited elevated mutational influence, potentially reflecting lineage-specific adaptation to extreme environments or demographic histories.

Overall, the observed compositional uniformity and lack of GC12-GC3 correlation suggest that functional constraints on protein structure operate largely independently of synonymous site evolution. These findings align with patterns reported in other angiosperm chloroplast genomes and reinforce that natural selection, which is driven by translational efficiency, tRNA availability, and fitness-related factors, is the predominant evolutionary force shaping codon usage in Cypripedioideae chloroplast genomes.

### 3.6 Ka/Ks analysis

**Figure 6:**
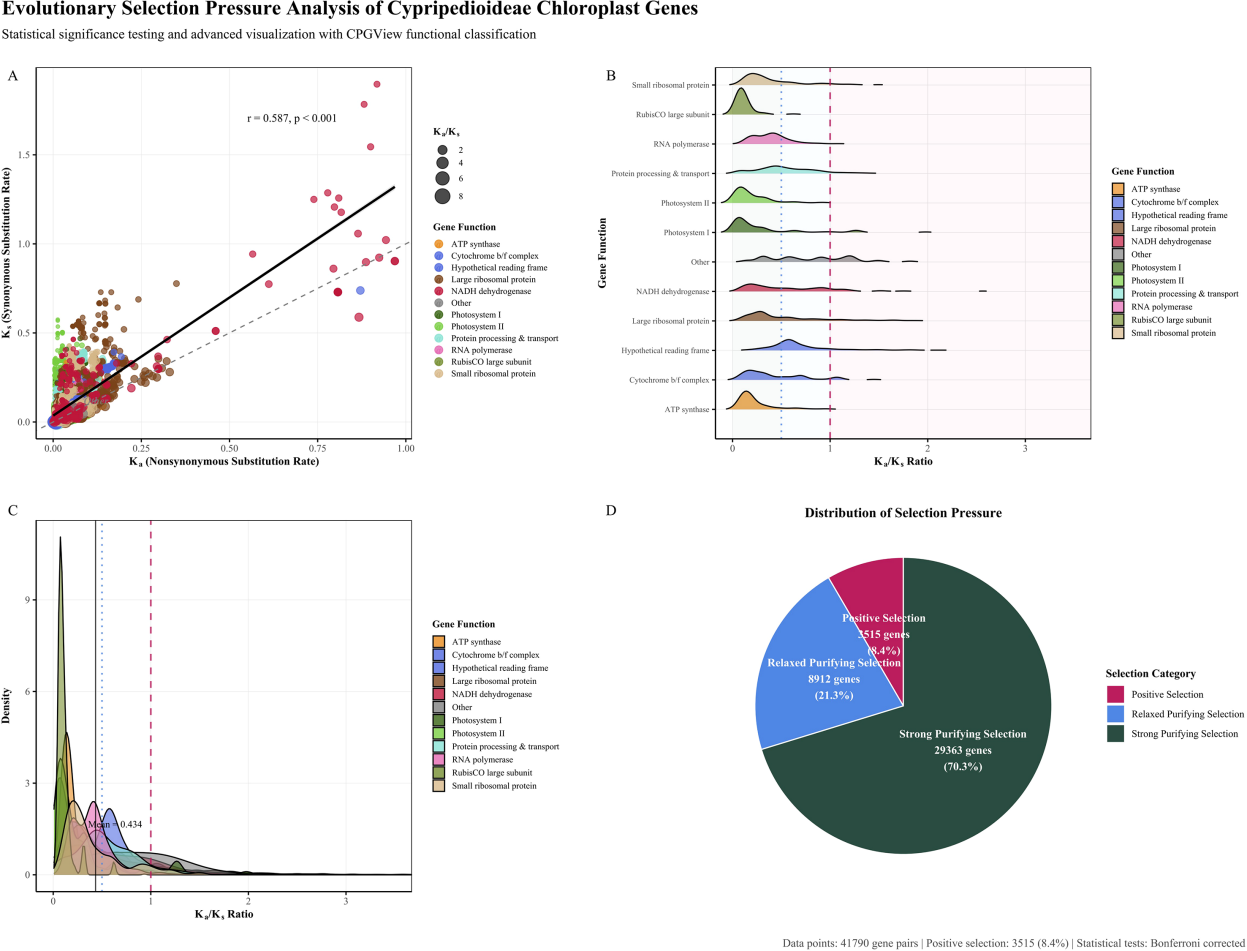
Evolutionary selection pressure analysis of Cypripedioideae chloroplast genes. **(A)** Scatter plot of Ka versus Ks rates colored by gene functional categories. Point size reflects Ka/Ks ratio magnitude. Diagonal dashed line indicates neutral evolution (Ka = Ks). **(B)** Ridge density plots showing Ka/Ks ratio distributions across gene functional categories. **(C)** Density curves of Ka/Ks ratios by gene functional categories with overall mean indicated. Vertical lines mark selection pressure thresholds at Ka/Ks = 0.5 and 1.0. **(D)** Pie chart showing the distribution of selection pressure categories across all analyzed gene pairs. Statistical significance testing performed with Bonferroni correction for multiple comparisons.

To assess evolutionary selection pressures on chloroplast protein-coding genes across Cypripedioideae, we calculated the ratio of nonsynonymous (Ka) to synonymous (Ks) substitution rates for 41,790 orthologous gene pairs from 48 species (**Figure 6D**). The Ka/Ks ratio serves as a standard indicator of selection intensity: values below 1 indicates purifying selection, near 1 suggests neutral evolution, and above 1 imply positive or diversifying selection. Ka and Ks estimates were obtained using a maximum likelihood framework, with multiple testing correction applied via the Bonferroni method. Genes were grouped into 12 functional categories to evaluate differential selective pressures across chloroplast metabolic pathways.

Purifying selection was the predominant evolutionary force acting on Cypripedioideae chloroplast genomes. Across all gene pairs, 91.6% exhibited Ka/Ks < 1.0, with 58.9% under strong purifying selection (Ka/Ks < 0.5) and 32.6% under relaxed purifying selection (0.5 ≤ Ka/Ks ≤ 1.0). Only 8.4% of gene pairs had Ka/Ks > 1.0, suggesting episodic positive selection or relaxed functional constraints (**Figure 6A**). A Ka versus Ks scatter plot revealed that core photosynthetic genes clustered in the low Ka-low Ks region, reflecting strong functional conservation. Regression analysis indicated a positive correlation between Ka and Ks, consistent with shared mutational processes and potential effects of generation time on evolutionary rates.

Selection pressure varied substantially among functional categories (**Figures 6B, C**; **Supplementary Table S6B**). The RubisCO large subunit (*rbcL*) exhibited the strongest purifying selection, with a mean Ka/Ks of 0.113, significantly lower than the subfamily average, and no gene pair showed evidence of positive selection. Photosystem II genes and ATP synthase subunits also experienced strong purifying selection, with mean Ka/Ks values of 0.213 and 0.263, respectively. In contrast, photosystem I genes displayed higher Ka/Ks ratios (mean = 0.308), with nearly 10% of pairs showing Ka/Ks > 1.0, potentially reflecting adaptive evolution in light-harvesting components. RNA polymerase genes were under moderate purifying selection (mean Ka/Ks = 0.410), whereas ribosomal proteins, particularly those of the large subunit, showed more relaxed constraints (mean Ka/Ks = 0.589).

NADH dehydrogenase genes, analyzed in a reduced subset of species due to frequent pseudogenization, had a mean Ka/Ks of 0.603. Hypothetical reading frames (*ycf* genes) and other genes, including *matK*, *clpP*, and *infA*, displayed the highest Ka/Ks values (0.811 and 0.800, respectively), indicative of either relaxed purifying selection or lineage-specific adaptive evolution. Wilcoxon rank-sum tests confirmed significant differences in selection pressure among functional categories, with the largest contrasts observed between small ribosomal proteins and photosystem II genes (**Supplementary Table S6C**). RubisCO consistently demonstrated the strongest purifying selection across all comparisons.

Correlation analyses revealed strong positive relationships between Ka and Ks within several gene groups, including *rbcL*, *ycf* genes, NADH dehydrogenase, and RNA polymerase, suggesting shared evolutionary constraints (**Supplementary Table S6D**). In contrast, weaker correlations in photosystem genes imply greater heterogeneity in selective pressures, potentially linked to ecological adaptations and variable light environments across species. Collectively, these results highlight the predominance of purifying selection in maintaining chloroplast gene function, while also identifying specific loci subject to lineage-specific adaptive evolution in Cypripedioideae.

### 3.7 Chloroplast genome collinearity and structural analysis

**Figure 7:**
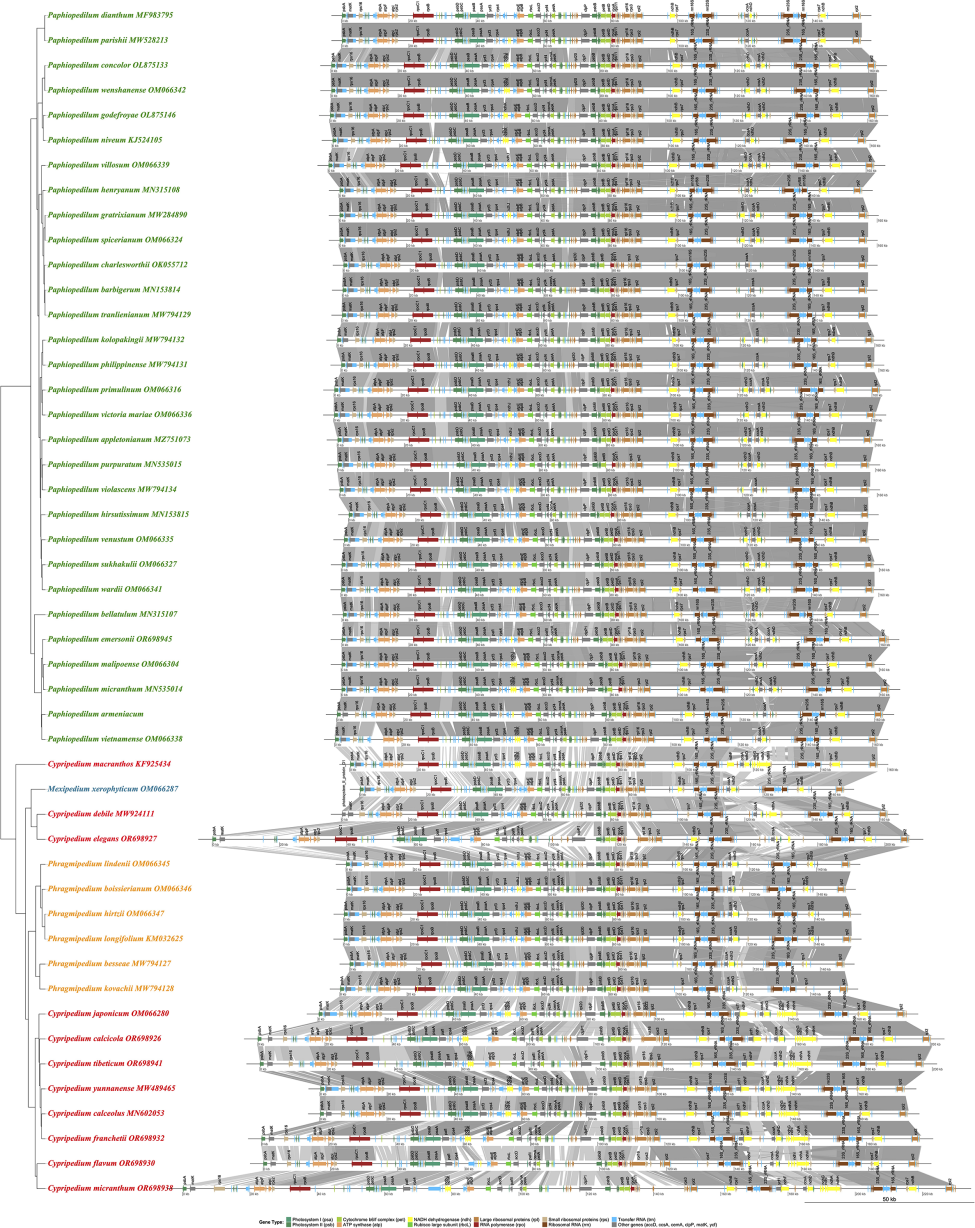
Comprehensive comparative analysis of chloroplast genome organization and gene synteny across 48 Cypripedioideae species. This phylogenetic comparison displays chloroplast genome structure, gene arrangement, and syntenic relationships among subfamily Cypripedioideae. The vertical gene arrangement panels show the relative position and orientation of each gene within the chloroplast genome. This comprehensive visualization reveals conserved gene order and synteny across the Cypripedioideae subfamily, while highlighting genus-specific and species-specific genomic rearrangements. The analysis demonstrates high conservation of chloroplast genome structure across the 48 species, with most variation concentrated in the inverted repeat boundaries and intergenic regions.

Genome-wide collinearity analysis across 48 Cypripedioideae species (30 *Paphiopedilum*, 11 *Cypripedium*, 6 *Phragmipedium*, and 1 *Mexipedium*) revealed largely conserved synteny interspersed with genus-specific structural rearrangements that delineate major evolutionary lineages within the subfamily (**Figure 7**, **Supplementary Table S7A**). All species maintained the canonical quadripartite plastome structure typical of angiosperms, consisting of LSC, SSC, and IR regions. Photosynthesis-related gene content and order were highly conserved, particularly across the LSC. Core photosynthetic genes, including *psbA*, *psaA*, *psaB*, and *rbcL*, ATP synthase subunits (*atpA*, *atpB*, *atpE*, *atpF*, *atpI*), and RNA polymerase genes (*rpoB*, *rpoC1*) maintained stable positional relationships, forming dense collinear blocks across the subfamily. These conserved blocks reflect strong functional constraints and correspond closely with phylogenetic signal, with species clustering according to taxonomic boundaries and shared genomic architecture.

Despite overall syntenic conservation, substantial heterogeneity was observed at junctions between genomic compartments, particularly at IR-SSC boundaries. These regions exhibited genus-specific dynamics that significantly contribute to genome size variation. *Paphiopedilum* species displayed the most pronounced boundary shifts, characterized by extensive IR expansion coupled with extreme SSC contraction, in some cases to as little as 524 bp, which is well below typical orchid values. This expansion resulted in the incorporation of several SSC-localized genes, including *ycf1*, *psaC*, *ndhD*, and *ccsA*, into the IRs. Considerable intrageneric variation in SSC structure was evident, particularly among *subgenus Parvisepalum*, suggesting ongoing plastome reorganization within the genus. The reference species *P. armeniacum* exemplifies this pattern, with genes normally confined to the SSC now located within IRs, highlighting evolutionary plasticity in this genomic region.

In contrast, *Cypripedium* plastomes expanded primarily through LSC enlargement, driven by proliferation of AT-rich repetitive sequences rather than IR boundary shifts. Genome sizes reached sizes up to 212,668 bp in *C. subtropicum*, among the largest reported in Orchidaceae. IR-SSC boundaries remained highly stable in *Cypripedium*, with LSC-IRb junctions consistently located near *rpl22* and SSC ends anchored at *ycf1*, in stark contrast to the dynamic boundaries in *Paphiopedilum*. Three *Cypripedium* species (*C. tibeticum*, *C. yunnanense*, and *C. formosanum*), exhibited a large-scale inversion of 75 kb within the LSC, spanning from *trnG-UCC* to *trnP-UGG*. These inversions, visible as reversed gene orientations in collinearity plots, likely arose independently in different taxonomic sections rather than from a common ancestor.

*Phragmipedium* and *Mexipedium* occupied an intermediate structural position, showing moderate IR expansion and SSC contraction less extreme than in *Paphiopedilum*, while maintaining compact genome sizes ranging from 147,384 bp to 151,238 bp. These genera formed a Neotropical sister clade to *Paphiopedilum* in phylogenetic analyses. Lineage-specific evolutionary signatures were also evident in *ndh* gene complements: all *Paphiopedilum* species lacked functional *ndhA*, *ndhE*, *ndhF*, *ndhG*, *ndhH*, and *ndhI* genes, with remaining *ndh* members present only as pseudogenes or entirely absent. In contrast, several *Cypripedium* species retained complete or partially functional *ndh* sets, including *ndhB*, *ndhJ*, and *ndhK*, indicating that *ndh* degradation occurred after the divergence of *Cypripedium* from other Cypripedioideae lineages. The correlation between IR-SSC boundary shifts and *ndh* gene loss suggests that structural rearrangements may have facilitated functional degradation of this gene family in *Paphiopedilum* and *Phragmipedium*.

Functional analysis further revealed that genes involved in photosynthesis, including photosystem subunits, cytochrome b_6_f complex components, and ATP synthase exhibited exceptional positional conservation with minimal structural variation. The *rbcL* gene displayed absolute syntenic stability, underscoring strong purifying selection on this essential carbon-fixing enzyme. Collectively, these collinearity patterns correlate strongly with both phylogenetic relationships and functional constraints across this ecologically and structurally diverse orchid subfamily.

### 3.8 Phylogenetic analysis

**Figure 8:**
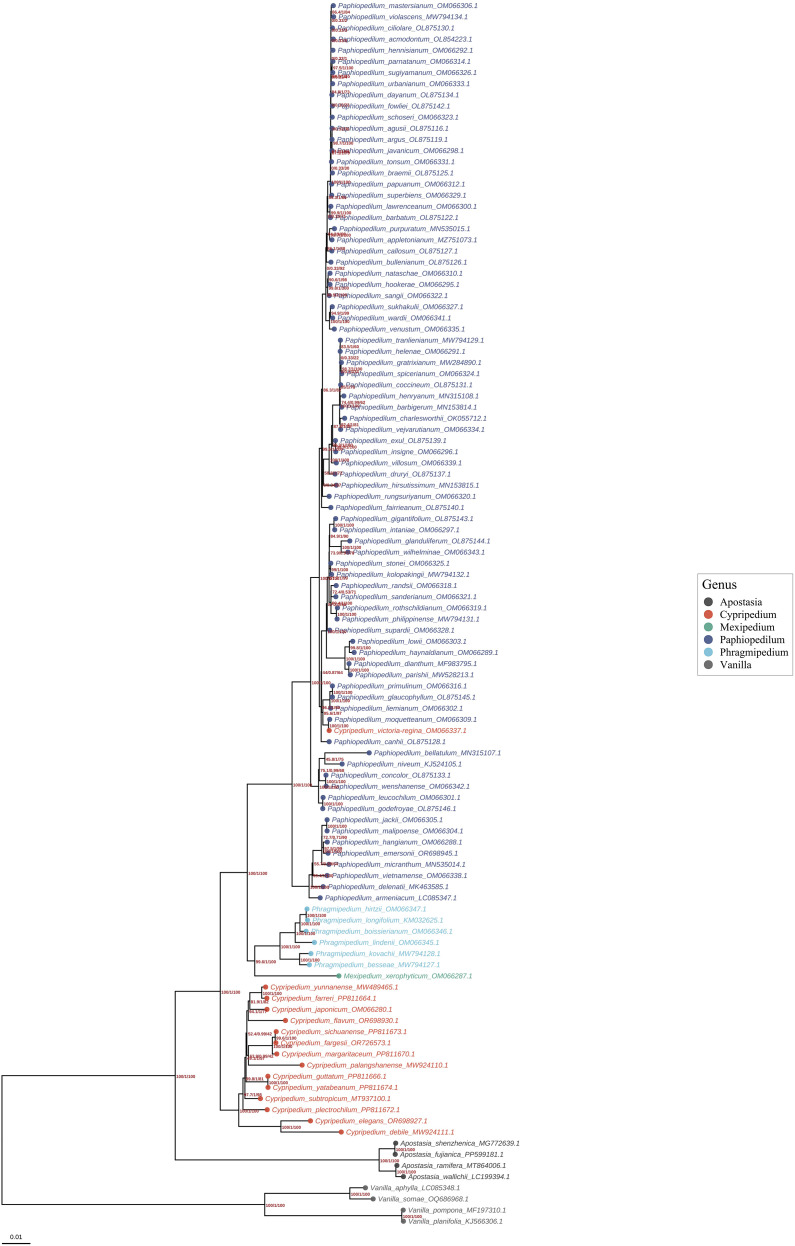
Maximum likelihood phylogeny of Orchidaceae based on complete chloroplast proteomes reveals subfamily relationships and evolutionary rate heterogeneity. The phylogenetic tree encompasses 233 species representing 47 genera across all five orchid subfamilies, with particular emphasis on Cypripedioideae. Branch tips are color-coded by genus, and circles at nodes indicate bootstrap support values.

Maximum likelihood phylogenetic reconstruction based on 81 concatenated chloroplast protein-coding genes from 110 Orchidaceae species robustly resolved intersubfamilial relationships and clarified the evolutionary position of the 102 studied Cypripedioideae taxa (**Figure 8**; **Supplementary Tables S8A, B**). The analysis included representatives from three orchid subfamilies: Apostasioideae, Vanilloideae, and Cypripedioideae, providing appropriate outgroup sampling for rooting the Cypripedioideae phylogeny. The Cypripedioideae formed a strongly supported monophyletic clade (UFBoot = 100%, SH-aLRT = 100%) sister to the Orchidoideae–Epidendroideae lineage, consistent with prior molecular systematic frameworks derived from nuclear and plastid phylogenomics. Among the 109 internal nodes in the tree, 107 received support values, with a mean UFBoot of 84.09% and a median of 100%. Notably, 66 nodes (60.6%) achieved strong support (UFBoot ≥95%), and 57 nodes (52.3%) received maximum support (UFBoot = 100%), indicating robust resolution of most relationships within the dataset. Within Cypripedioideae, four major generic lineages were recovered with high statistical confidence: *Cypripedium* (15 species), *Paphiopedilum* (80 species), *Phragmipedium* (6 species), and *Mexipedium* (1 species), each reflecting distinct evolutionary trajectories. *Cypripedium* occupied the most basal phylogenetic position within the subfamily, consistent with its retention of plesiomorphic chloroplast genome features including larger genome sizes and functional *ndh* gene complements. Phylogenetic structuring within *Cypripedium* corresponded to biogeographic patterns, with distinct clades comprising East Asian taxa (*C. japonicum*, *C. formosanum*), Eurasian species (*C. calceolus*), and high-elevation Himalayan lineages (*C. tibeticum*, *C. flavum*). Branch length heterogeneity among *Cypripedium* species suggests differential molecular evolutionary rates potentially associated with adaptation to diverse thermal and elevational gradients. *Paphiopedilum* was recovered as a strongly monophyletic genus (UFBoot = 100%), exhibiting markedly more uniform evolutionary rates compared to *Cypripedium*. Infrageneric topology was largely congruent with established morphology-based subgeneric classifications. Species from subgenus *Parvisepalum*, including *P. armeniacum*, *P. micranthum*, *P. emersonii*, and *P. malipoense*, formed a cohesive high-elevation clade characterized by short internal branches and limited molecular divergence, consistent with recent diversification in montane habitats of southwestern China and northern Vietnam. *Phragmipedium* and *Mexipedium xerophyticum* formed a well-supported Neotropical sister clade, reflecting their shared biogeographic history in the Americas. This relationship was recovered with strong support (UFBoot = 98%), corroborating previous molecular studies suggesting vicariance of conduplicate-leaved Cypripedioideae between Old World (*Paphiopedilum*) and New World (*Phragmipedium* and *Mexipedium*) lineages. The eight outgroup species from Apostasioideae and Vanilloideae were correctly resolved as successive early-diverging lineages, with Apostasioideae sister to all remaining Orchidaceae and Vanilloideae sister to the Cypripedioideae, Orchidoideae, Epidendroideae clade. This topology is consistent with the current understanding of orchid subfamily relationships based on comprehensive phylogenomic analyses. Integration of phylogenetic topology with genomic and structural data revealed significant associations between evolutionary divergence and chloroplast genome architecture. The basal position of *Cypripedium* corresponds to its distinctive expanded plastomes and retention of functional *ndh* genes, whereas the derived *Paphiopedilum* clade exhibits contracted genomes with extensive IR expansion, SSC contraction, and complete *ndh* gene loss. These patterns indicate that plastome architectural evolution accompanied and potentially facilitated lineage diversification within Cypripedioideae, providing molecular signatures that correlate with ecological specialization across temperate, subtropical, and tropical habitats.

### 3.9 Multi-group comparison of chloroplast genome characteristics

**Figure 9:**
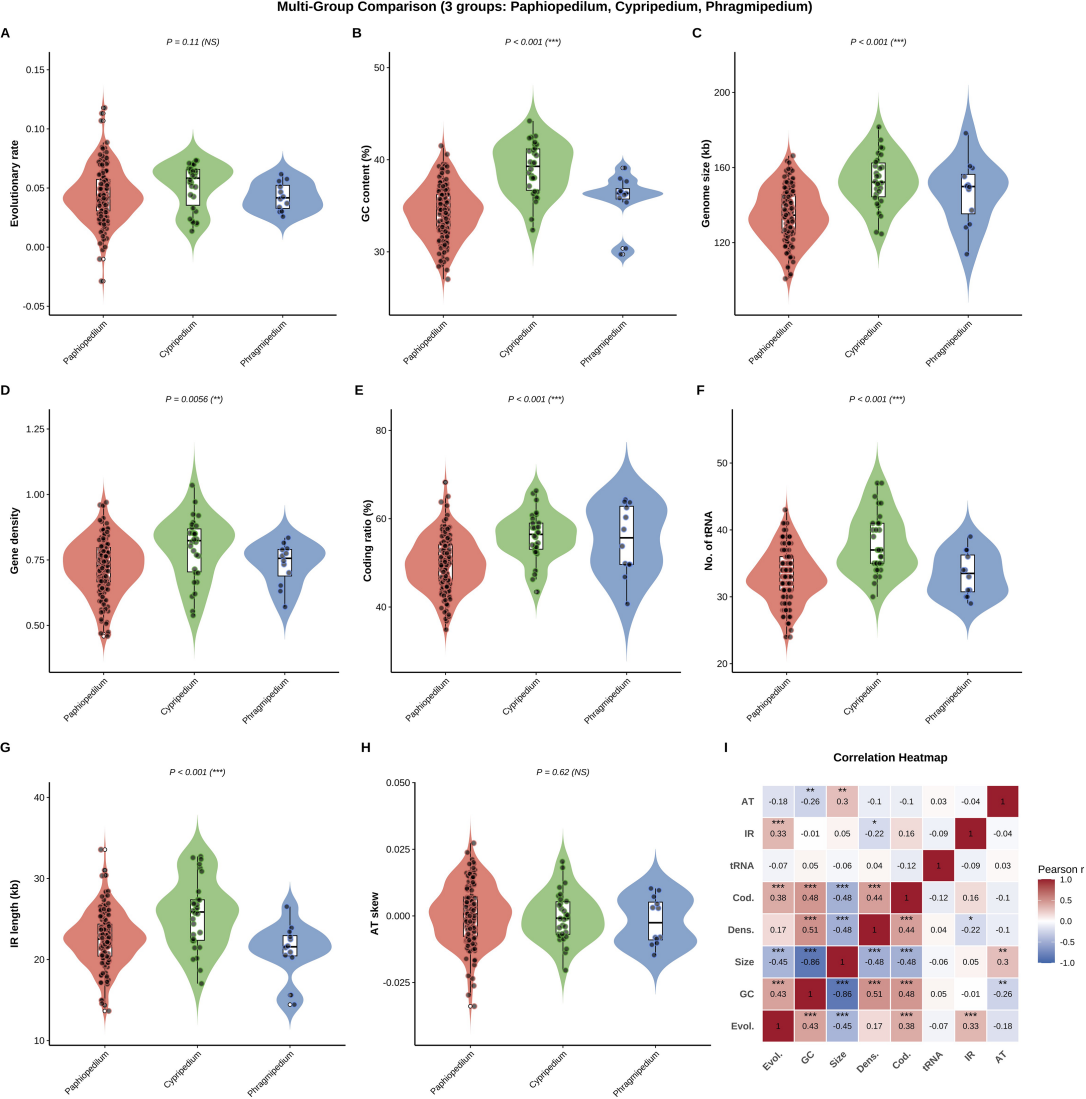
Multi-group statistical comparison of chloroplast genome characteristics across three Cypripedioideae genera. **(A–H)** Violin plots with embedded boxplots comparing eight genomic parameters among *Paphiopedilum*, *Cypripedium*, and *Phragmipedium*. Individual data points are overlaid as jittered scatter. P-values from Kruskal-Wallis tests are shown above each panel with significance levels indicated as: P < 0.001, P < 0.01, NS = not significant. **(A)** Evolutionary rate (substitutions/site). **(B)** GC content (%). **(C)** Genome size (kb). **(D)** Gene density (genes/kb). **(E)** Coding ratio (%). **(F)** Number of tRNA genes. **(G)** Inverted repeat length (kb). **(H)** AT skew. **(I)** Pearson correlation heatmap showing pairwise relationships among all eight parameters. Correlation coefficients are displayed within cells, with significance levels indicated as asterisks (P < 0.05, P < 0.01, P < 0.001). Color scale ranges from blue (negative correlations) through white (no correlation) to red (positive correlations).

Comparative analysis revealed significant divergence in chloroplast genome features among the three Cypripedioideae genera (*Cypripedium*, *Paphiopedilum*, *Phragmipedium*). Six of the eight parameters examined showed statistically significant inter-generic variation (**Figures 9A–H**, **Supplementary Table S9**). While the mean evolutionary rate did not differ significantly (P = 0.11), *Cypripedium* exhibited the highest mean rate. Significant differences were detected in GC content (P < 0.001), with *Cypripedium* possessing the highest value. Genome size also varied significantly (P < 0.001), being largest in *Cypripedium* and most compact in *Paphiopedilum*. Gene density and the proportion of coding sequence both differed among genera (P = 0.0056 and P < 0.001, respectively), with *Cypripedium* showing the highest values. The number of tRNA genes and IR length were also significantly different (both P < 0.001), with *Cypripedium* possessing the highest counts/lengths. In contrast, AT skew was conserved across genera (P = 0.62). Correlation analysis indicated strong interdependencies among genomic parameters (**Figure 9I**). Genome size was negatively correlated with GC content (r = −0.86, P < 0.001). Evolutionary rate showed positive correlations with GC content and gene density. The coding ratio was positively correlated with gene density and negatively with genome size. IR length demonstrated only weak correlations with other features.

## 4 Discussion

This study presents a comprehensive analysis of CUB and structural variation across 48 complete Cypripedioideae chloroplast genomes. Our results indicate that codon usage bias is predominantly shaped by natural selection rather than mutational pressure, driving lineage-specific molecular adaptations across diverse ecological niches. Although the overall plastome architecture is largely conserved, notable species-specific variation occurs in key regions, particularly at inverted repeat (IR) boundaries. These findings provide novel insights into the evolutionary dynamics of plant chloroplast genomes and their role in facilitating ecological adaptation (Sheng et al., 2021; Tang et al., 2023).

### 4.1 Chloroplast genome size and structural characteristics

Our comprehensive analysis of 48 Cypripedioideae species revealed substantial chloroplast genome size variation within this subfamily. Plastome sizes ranged from 147,384 to 229,932 bp, markedly exceeding the terrestrial plant average of ~151 kb, while GC content varied from 26.66% to 37.82%, diverging from the typical angiosperm average of ~37.6%. These differences correspond to phylogenetic lineages and their associated ecological niches. For instance, *Cypripedium* species, largely distributed across temperate and alpine regions of the Northern Hemisphere, exhibited an average plastome size of 188,297 bp, significantly larger than that of tropical and subtropical *Paphiopedilum* species (averaging 159,016 bp). This expansion in *Cypripedium* is primarily attributable to the proliferation of AT-rich repetitive sequences in noncoding regions, particularly intergenic spacers (IGS) and introns within the LSC region. Additionally, *Cypripedium* plastomes display lower mean GC content of 32.01%, compared to 35.70% in *Paphiopedilum*, potentially reflecting adaptive responses to contrasting thermal regimes, analogous to patterns reported in temperature-adapted Theaceae species (Wang et al., 2022) and high-elevation *Saxifraga* species. *Phragmipedium* species exhibit intermediate genomic features, with a mean genome size of 149,927 bp and GC content of 36.21%, situating them between *Cypripedium* and *Paphiopedilum* in plastome architecture. The observed correlation between genome architecture and phylogenetic distance mirrors trends in other angiosperms (Li et al., 2023).

Cypripedioideae plastomes retain the canonical quadripartite structure comprising LSC, SSC, and paired IR regions. However, IR/SSC boundary regions exhibit pronounced species-specific variation correlating with phylogenetic lineages, a pattern increasingly recognized in Orchidaceae. Substantial differences in gene content and positional dynamics occur across the four junction regions (JLB, JSB, JSA, and JLA) among the examined taxa. For example, in *P. venustum*, the *rpl22* gene is entirely contained within the JLB boundary and is notably longer than in congeners, whereas in the reference species *P. armeniacum*, *ycf1* spans JSB and JSA boundaries. Similar IR boundary dynamics are observed in *Cycas* (Tang et al., 2023) and *Pinus*, suggesting that such variation is a shared evolutionary feature across seed plants. Comparable patterns in Cyanobacteria further indicate that IR boundary dynamism may be a conserved mechanism across prokaryotic and eukaryotic lineages (Hagemann et al., 2016). Phylogenomic evidence demonstrates that IR shifts often occur independently across plant lineages, suggesting convergent evolution rather than shared ancestry (Wicke et al., 2011). Within Cypripedioideae, IR expansion and contraction patterns vary among phylogenetic clades with similar IR architectures reflecting closer evolutionary affinities, while divergent boundaries indicate substantial evolutionary divergence, consistent with observations in *Malus* and *Prunus* species (Li et al., 2023) These findings highlight the potential utility of IR boundary variation as a phylogenetic and evolutionary marker.

At the functional gene level, core photosynthetic genes (*rbcL*, *psbA*, *psaB*) are highly conserved across all examined Cypripedioideae species, exhibiting strong purifying selection (Ka/Ks < 0.15). In contrast, genes involved in environmental responses (*matK*, *ycf1*) exhibit higher variability and elevated Ka/Ks ratios, potentially reflecting adaptive evolution or relaxed functional constraints throughout subfamily. These patterns align with observations in other lineages, including *Gnetales* (Yang et al., 2024) and *Magnoliaceae* (Geng et al., 2022), underscoring the role of functional gene conservation and selective pressures in shaping plastome evolution. Despite these insights, we feel that functional validation linking IR boundary variation to gene expression or physiological performance remains unexplored. Future work could integrate plastome transcriptomics, proteomics, or plastid transformation to directly assess the adaptive consequences of IR expansions/contractions and gene-level variation across ecological gradients. Additionally, expanding taxon sampling within underrepresented lineages may reveal finer-scale evolutionary dynamics and the prevalence of convergent IR boundary shifts.

### 4.2 Codon usage bias patterns and their ecological correlates

The chloroplast genomes of the 48 analyzed Cypripedioideae species exhibited pronounced nonrandom codon usage bias with a strong bias for A/U-ending codons over G/C-ending codons at synonymous third positions. Among the six leucine-encoding synonymous codons, UUA and UUG displayed mean RSCU values of 1.849 and 1.248, respectively, substantially exceeding those of CUA, CUC, CUG, and CUU. This AT-biased codon usage is consistent with patterns observed in most seed plants, including several angiosperm lineages such as Theaceae, Rutaceae, and *Solanum*, as well as gymnosperms like Gnetales (Yang et al., 2024). Regression analyses of GC12 against GC3 revealed minimal correlation, indicating that mutational pressure contributes only marginally to codon usage patterns, while natural selection predominantly shapes synonymous codon choice.

Environmental context appears to drive lineage-specific codon optimization in Cypripedioideae (Hu et al., 2022). High-altitude *Cypripedium* species exhibited stronger A/T-ending codon preferences than low-altitude *Paphiopedilum*, with mean RSCU values of 1.56 versus 1.41, respectively. Notably, high-elevation species, such as *C. tibeticum*, showed pronounced codon usage bias in genes associated with cold tolerance, reflected by ENC values on average 5.2 units lower than those in low-altitude species. This indicates intensified selection for specific synonymous codons, likely promoting translational efficiency and accuracy under cold stress. Conversely, tropical *Paphiopedilum* species, including *P. villosum* and *P. delenatii* exhibited distinctive codon usage in genes potentially linked to humidity adaptation highlighting the influence of ecological pressures on plastome evolution. In *P. armeniacum*, a subtropical montane orchid, natural selection was inferred to account for approximately 92.4% of codon usage variation, supported by a mean ENC of 52.28 and a neutrality slope of 0.0763. These patterns underscore the role of codon usage as a fine-tuning mechanism for physiological optimization in response to environmental constraints, in line with findings in climatically diverse Theaceae species (Wang et al., 2022).

Functional categories of genes display differential codon usage bias. Core photosynthesis-related genes (*rbcL*, *psbA*, and *atpA*) showed strong codon conservation across all 48 species, indicative of purifying selection maintaining essential chloroplast functions. In contrast, genes associated with environmental adaptation such as *matK* and *ndhF* exhibited pronounced species-specific variation, consistent with lineage-specific adaptive evolution. Integration with Ka/Ks analyses, which revealed elevated nonsynonymous substitution rates in several adaptive genes, indicate that selective pressures vary among functional categories, driving differential codon optimization across the plastome. Similar gene-specific patterns of functional divergence in codon usage have been reported in *Cistanche* (Feng et al., 2023), reinforcing the concept that codon usage evolution is modulated by selective constraints at the functional level.

Although overall codon usage trends were broadly conserved across the *Cypripedium*, *Paphiopedilum*, *Phragmipedium*, and *Mexipedium* genera, specific codon frequency differences were substantial, potentially reflecting divergent evolutionary histories following lineage separation. Phylogenetically proximate genera displayed more congruent codon usage patterns, with correlation coefficients of 0.94 between *Phragmipedium* and *Paphiopedilum* reaching 0.94 versus 0.87 with *Cypripedium*, highlighting the utility of codon usage bias as a complementary molecular phylogenetic marker (Chakraborty et al., 2020). While codon usage patterns suggest adaptation to environmental pressures, functional validation remains lacking. We suggest that future research could employ plastid transformation or in vitro translational assays to test whether preferential A/U-ending codons enhance protein synthesis efficiency under specific stress conditions. Comparative analyses linking codon bias to tRNA availability and translational efficiency across species and environments could further clarify mechanistic bases for adaptive codon optimization. Expanding taxonomic coverage to include additional Cypripedioideae and related orchid species could also reveal lineage-specific innovations and refine the role of codon usage as a marker of ecological adaptation.

### 4.3 Phylogenetic relationships and genome structure evolution

Phylogenetic reconstruction based on complete chloroplast genome sequences from 48 Cypripedioideae species robustly resolved genus-level differentiation and intrageneric clustering, confirming the subfamily’s monophyly. *Cypripedium* forms a distinct basal lineage, sister to a *Paphiopedilum-Phragmipedium* clade, which further subdivides into East Asian, Eurasian, and North American branches. This concordance between geographic distribution and phylogenetic structure suggests that evolutionary history is closely tied to ancient continental separation and Cenozoic climatic shifts (Kissling et al., 2012). Significant correlations were observed between interspecific phylogenetic relationships and chloroplast genome structural characteristics (Wang et al., 2021), with IR boundary variation and gene rearrangement patterns showing clade-specific signatures, These findings indicate that plastome architectural evolution is an active driver of lineage differentiation and speciation, consistent with trends documented in other orchid lineages (Zhang et al., 2022).

Comparative analyses of morphologically similar yet geographically disjunct species highlight complex evolutionary dynamics (Varga and Schmitt, 2008). For example, despite floral convergence between *C. tibeticum* and *C. macranthos*, their placement in distinct clades indicates that morphological similarity arises through convergent evolution rather than shared ancestry. Conversely, morphologically divergent yet phylogenetically proximate species, such as *C. japonicum* and *C. formosanum*, demonstrate associations between lineage-specific morphological innovations and historical evolutionary events. Within *Paphiopedilum*, species occupying distinct ecotypes show phylogenetic clustering reflecting environmental adaptation; alpine-adapted species (*P. armeniacum* and *P. emersonii*) form a cohesive cluster within subgenus *Parvisepalum*, separating them from lowland tropical congeners. These patterns underscore the intimate link between ecological pressures, phylogenetic history, and plastome structural variation.

Integrated phylogenetic and structural analyses of chloroplast genomes reveal that diversification in Cypripedioideae is tightly coupled with plastome architectural evolution. Genomic size variation is pronounced, with *Cypripedium* exhibiting expanded genomes averaging 188,297 bp, compared to the more compact plastomes of *Paphiopedilum* averaging 159,016 bp. These differences reflect distinct evolutionary trajectories: *Cypripedium* lineages have accumulated extensive AT-rich repetitive sequences in noncoding regions, whereas *Paphiopedilum* has undergone pronounced inversion repeat expansion coupled with severe contraction of the small single-copy region. Additional mechanisms driving lineage differentiation include IR boundary mutations, progressive loss or pseudogenization of *ndh* genes, particularly evident in *Paphiopedilum* and *Phragmipedium*, and localized genomic rearrangements. Collectively, these structural variations have likely facilitated adaptive evolution and ecological diversification, enabling Cypripedioideae to radiate across alpine tundra, temperate forests, and tropical rainforest habitats (Guo et al., 2021a; Lin et al., 2015; Ringelmann and Wagner, 2025).

We believe that our new findings highlight the predictive power of plastome structure in resolving phylogenetic relationships and identifying adaptive traits. Structural features such as IR/SSC boundary dynamics and *ndh* gene retention offer molecular markers for lineage assignment and may inform conservation strategies by linking genomic architecture to ecological resilience. Moreover, the integration of phylogenetic, genomic, and structural data provides a framework for identifying candidate loci for functional and adaptive studies, including plastid-based transformation targets to enhance stress tolerance. But, while our study elucidates broad correlations between genome structure and ecological adaptation, functional validation of the adaptive significance of structural rearrangements remains unexplored. Future research could include: (1) experimental assays linking IR expansion or SSC contraction to transcriptional output or photosynthetic efficiency; (2) physiological studies assessing the impact of *ndh* gene loss on chlororespiration and stress response; and (3) population-level sampling to evaluate the role of structural polymorphisms in local adaptation. Additionally, expanding taxonomic coverage to other orchid subfamilies would allow comparative analyses to test whether structural-genomic evolution follows generalizable patterns across Orchidaceae.

## 5 Conclusions

Through comparative analyses of 48 complete plastomes, this study elucidates the molecular mechanisms underpinning underlying adaptive radiation in Cypripedioideae and advances our current understanding of orchid evolutionary biology. We demonstrate that synonymous codon usage is predominantly shaped by natural selection rather than mutational pressure, highlighting codon optimization as a functional driver of physiological and ecological adaptation. Moreover, lineage-specific plastome structural evolution including lineage-specific IR expansion, SSC contraction, accumulation of AT-rich repetitive elements, and differential *ndh* gene retention— closely correlates with ecological specialization. These findings reveal that chloroplast genome architecture not only reflects evolutionary history but also actively contributes to diversification, providing a mechanistic link between genomic structure and adaptive radiation. The integration of phylogenomic analyses, structural variation mapping, and codon usage patterns profiling offers a comprehensive framework that resolves major clades and uncovers how genomic traits track ecological gradients. Our results indicate that adaptive evolution in Cypripedioideae is jointly shaped by sequence-level optimization, functional gene content evolution, and large-scale structural rearrangements, demonstrating the interplay between genomic architecture, selective regimes, and phylogenetic history in driving evolutionary trajectories in flowering plants. The molecular signatures identified here, including lineage-specific codon preferences, IR/SSC boundary dynamics, and patterns of *ndh* gene retention, represent valuable resources for applied research. These features can serve as molecular markers for phylogenetic inference, guide conservation genomics initiatives, and inform functional studies. Understanding the adaptive significance of these genome-level traits can facilitate breeding programs, conservation strategies, and synthetic biology applications aimed at enhancing stress tolerance, photosynthetic efficiency, or environmental resilience in orchids and related plant species.

Despite these advances, the study also highlights several gaps and limitations. Although we establish strong correlations between genome structure, codon usage. and ecological adaptation, experimental validation is still limited. Future research should address the functional consequences of codon optimization, for example through laboratory assays or plastid transformation experiments to, determine whether specific A/U-ending codons improve translational efficiency or confer tolerance to abiotic stresses such as cold, drought, or low light. Similarly, the regulatory impacts of plastome structural shifts, remain largely unexplored. Comparative, expression profiling across environmental, gradients could reveal whether IR expansion or SSC contraction influences transcript abundance or physiological performance. The functional implications of *ndh* gene loss also require further investigation, as physiological and biochemical analyses could clarify whether these losses reflect adaptive metabolic reprogramming or simply relaxed selective constraints. Finally, broader taxonomic and population-level sampling across Orchidaceae, including multiple populations per species would allow testing of whether structural and codon usage variations contribute to local adaptation or broader patterns of adaptive radiation.

Collectively, we believe that our findings provide a robust genomic foundation for linking plastome architecture with functional adaptation and ecological diversification. By combining molecular, structural, and ecological perspectives, this study opens new avenues for both fundamental research into plant evolutionary biology and applied approaches in conservation breeding, and functional genomics, offering a deeper understanding of how genomic architecture shapes adaptation and diversification in flowering plants.

## Data Availability

The datasets presented in this study can be found in online repositories. The names of the repository/repositories and accession number(s) can be found in the article/**Supplementary Material**.
